# The first spontaneous resolution of a sulfoxide: Dianin’s compound analogue, (*R*)-4-(4-hy­droxy­phen­yl)-2,2,4-tri­methyl­thia­chroman-1-oxide

**DOI:** 10.1107/S2056989018014366

**Published:** 2018-10-19

**Authors:** James H. Gall, J. Derek White, David D. MacNicol, Christopher S. Frampton

**Affiliations:** a School of Chemistry, Joseph Black Building, University Avenue, University of Glasgow, Glasgow, G12 8QQ, Scotland; b Experimental Techniques Centre, Brunel University London, Kingston Lane, Uxbridge, UB8 3PH, England

**Keywords:** crystal structure, *thia*-Dianin’s compound, hydrogen bonding, spontaneous resolution

## Abstract

The title sulfoxide, C_18_H_20_O_2_S, was prepared by controlled oxidation of *thia*-Dianin’s compound using hydrogen peroxide in glacial acetic acid. On recrystallization from glacial acetic acid, it was found to form unsolvated, spontaneously resolved crystals, the initial crystal structure analysis revealing the presence of both sulfoxide epimers in the crystal. On multiple recrystallization a single epimer was observed, with crystallization occurring in the unchanged ortho­rhom­bic space group *P*2_1_2_1_2_1_, with *Z′* = 1. The mol­ecule possesses a *distal* conformation, referring to the juxtaposition of the *p*-hydoxyphenyl substituent with respect to its *syn-*related methyl group, with the sulfoxide oxygen atom *anti* to the aromatic substituent. The mol­ecular packing features O—H⋯O hydrogen bond chains running parallel to the *b* axis of the unit cell.

## Abstract 2

The single epimer of Dianin’s compound analogue, (*R*)-4-(4-hy­droxy­phen­yl)-2,2,4-tri­methyl­thia­chroman-1-oxide, was prepared by multiple recrystallization and studied by X-ray diffraction.

## Chemical context   

A significant body of work in the literature relates to specific­ally targeted structural modification of Dianin’s compound, 4-(4-hy­droxy­phen­yl)-2,2,4-tri­methyl­chroman **2**, (MacNicol, 1984 ▸; Finocchiaro & Failla, 1996 ▸; Collet & Jacques, 1976 ▸; Frampton *et al.*, 2017*a* ▸,*b* ▸,*c* ▸). Crystallization of the new compounds has normally resulted in one of two outcomes: formation of clathrates in the space group *R* 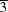 (or *R*3) or spontaneous resolution, also a subject of much current inter­est (Pérez-García & Amabilino, 2007 ▸), to form an unsolvated conglomerate in space group *P*2_1_2_1_2_1_, with *Z′* = 1, in which the individual crystals are formed by supra­molecular assembly of a single enanti­omer. A notable departure from the above crystallization modes has, however, been found in the case of Dianin’s sulfone **4**, (Frampton *et al.*, 1992 ▸), which crystallizes unsolvated in the polar monoclinic space group *C*c, with *Z*′ = 1, and these crystals exhibited a significant SHG effect. The present work was undertaken to establish if the corresponding sulfoxide **1** would retain the clathrating ability of its immediate progenitor *thia*-Dianin’s compound **3**, or would undergo spontaneous resolution, alternative possibilities being the formation of a polar monoclinic crystal or crystallization in a more frequently encountered space group. Inter­estingly, the achiral bis-sulfoxide *trans-*(*R,S*)-α,α′-di-*tert*-butyl­sulfinyl-*para*-xylene undergoes conformational spontaneous resolution in the space group *P*2_1_2_1_2_1_: on dissolution, rapid conformational racemization occurs at room temperature; however, the authors make the point that at 173 K, from calculations, it could be possible to obtain one chiral conformation from a single crystal (Xu *et al.*, 2014 ▸). Accordingly, the sulfoxide **1** was prepared by controlled oxidation of **3** as described in Section 5, and its crystal structure determined.
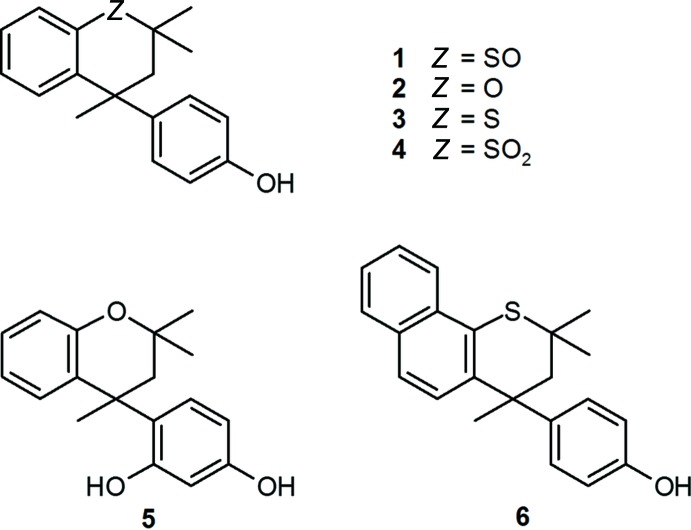


## Structural commentary   

**Figure 1:**
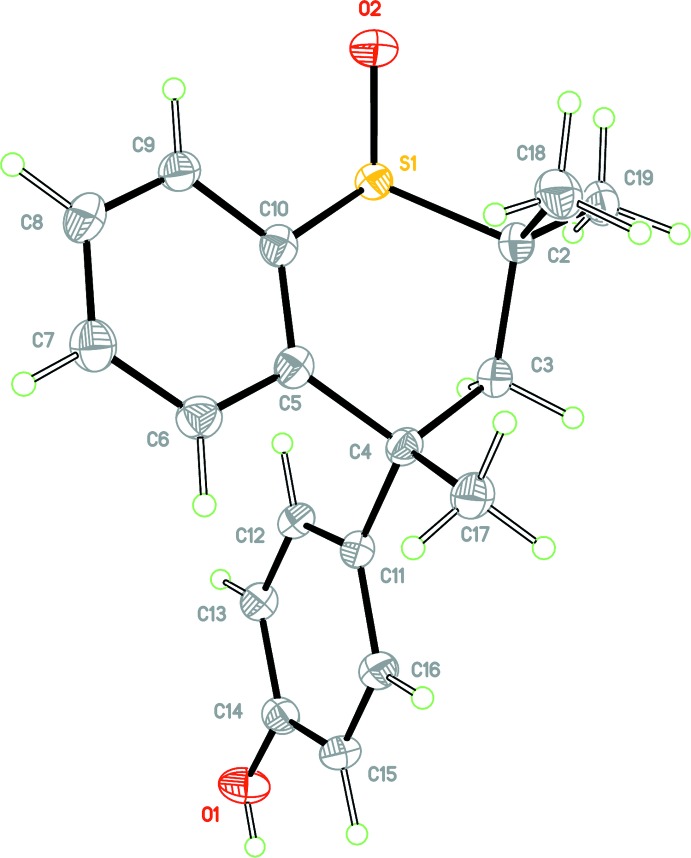
View of mol­ecule **1** with the atom-labelling scheme. Ellipsoids are drawn at the 50% probability level.

**Figure 2:**
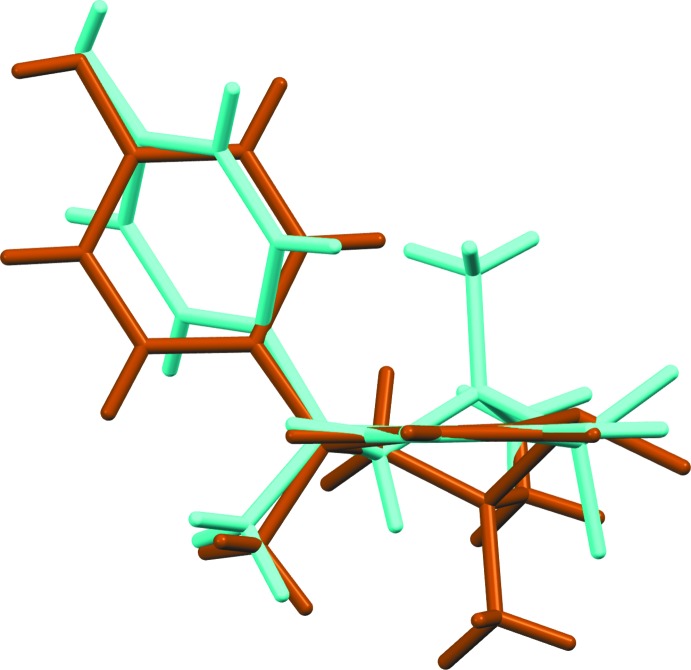
View of the structure overlay of **1** (brown) and **4** (cyan).

Initial attempts to determine the crystal structure of **1** revealed the presence of both sulfoxide epimers in the crystal in a ratio of approximately 90:10. It was found that multiple recrystallization of **1** from glacial acetic acid yielded a single epimer, the structure of which is presented here. The crystal structure of **1** is ortho­rhom­bic, space group *P*2_1_2_1_2_1_ with a single independent mol­ecule in the asymmetric unit, (*Z*′ = 1), Fig. 1 ▸. The mol­ecule possesses a *distal* conformation, this referring to the juxtaposition of the *p*-hy­droxy­phenyl subs­tit­uent with respect to the *syn-*related methyl group. The C2—C3—C4—C11 torsion angle is 154.0 (2)°, the corresponding torsion angle for racemic Dianin’s compound **2** has a magnitude of 80.67° (Lee *et al.*, 2014 ▸) and for 4-(4-hy­droxy­phen­yl)-2,2,4-tri­methyl­chroman-1,1-dioxide **4**, it is 76.8° (Frampton *et al.*, 1992 ▸). The expected torsional angle value for a *distal* conformation is 160° whereas that for a *proximal* conformation is 80°. The torsion angle S1—C2—C3—C4, defining the heterocyclic ring chirality, has a value of −67.3 (2)°. Fig. 2 ▸ shows an overlay of **1** (brown) with sulfone **4** (cyan). In this figure, the six aromatic atoms of the chroman unit for each structure have been overlaid using the standard mol­ecule overlay routine in *Mercury* (Macrae *et al.*, 2008 ▸), resulting in an r.m.s. displacement of 0.0147 Å, and this clearly demonstrates the difference between the *distal* and *proximal* conformations of **1** and **4**, respectively. The absolute configuration of **1**, was determined as being *R* at the chiral centre C4 by anomalous dispersion methods, (Parsons *et al.* 2013 ▸), the Flack *x* parameter was determined as −0.002 (7) using 1246 quotients [(*I* ^+^) − (*I* ^−^)]/[(*I* ^+^) + (*I* ^−^)].

## Supra­molecular features   

**Figure 3:**
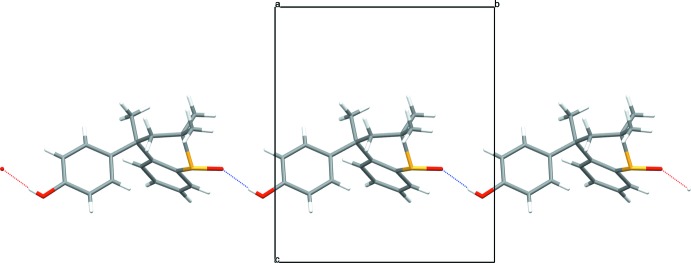
A partial view of the crystal packing down the *a* axis showing the hydrogen-bonded chain. The inter­molecular O—H⋯O hydrogen bond is shown as a dotted line.

The structure of **1** is isostructural with the enanti­omerically pure amine counterparts of Dianin’s and *thia*-Dianin’s compound, (*R*)-4-(4-amino­phen­yl)-2,2,4-tri­methyl­chroman and (*S*)-4-(4-amino­phen­yl)-2,2,4-tri­methyl­thia­chroman, both of which were obtained by spontaneous resolution (Frampton *et al.*, 2011 ▸), and also surprisingly isostructural with the enanti­omerically pure forms of 4-(4-hy­droxy­phen­yl)-2,2,4-tri­methyl­chroman, **2** (Lloyd & Bredenkamp, 2005 ▸) and 4-(2,4-di­hydroxy­phen­yl)-2,2,4-tri­methyl­chroman, **5** (Beresford *et al.*, 1999 ▸). The crystal packing is dominated by the formation of an extended linear hy­droxy –OH to sulfoxide O, hydrogen-bonded O—H⋯O chain along the [010] direction of the unit cell, Figs. 3 ▸ and 4 ▸, Table 1 ▸.

## Database survey   

A search of the Cambridge Structural Database (CSD, Version 5.39 update August 2018; Groom *et al.*, 2016 ▸) for the *thia*-Dianin’s framework, **3**, yielded 15 hits, all of which were genuine examples of analogues of the material under investigation. Although there are no entries for the empty racemic *R* 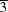 host of *thia*-Dianin’s compound, there are seven entries for the following host–guest clathrates: ethanol (HPTHCR; MacNicol *et al.*, 1969 ▸), 2,5,5-tri­methyl­hex-3-yn-2-ol (TCHHXO; MacNicol & Wilson, 1971 ▸), cyclo­pentane (METCCP; Hardy *et al.*, 1979 ▸) and *iso*propanol at four different temperatures demonstrating three commensurate phase changes in the host lattice (VANFOI, 371 K, VANFOI01, 295 K, VANFOI02, 200 K and VANFUO, 90 K; Frampton *et al.*, 2017*a* ▸). *Thia*-Dianin’s compound, **3**, was also found in the 1:1 quasi*-*racemic *R*3 host with Dianin’s compound, **2**, in the following three entries: apohost (BIBNAD and BIBNAD01), CCl_4_/H_2_O host–guest clathrate (HIDQAO) (Frampton *et al.*, 2013 ▸). The structure and absolute stereochemistry determination of the resolved *S*-enantio­mer of *thia*-Dianin’s compound used in the formation of the quasi-racemates above (BIBNEH: Frampton *et al.*, 2013 ▸). Four further examples demonstrating a slightly modified framework include the 6-methyl analogue (HPMTCM; Hardy *et al.*, 1977 ▸), the cyclo­ctane host–guest clathrate of the 8-methyl analogue (MSOCYO10; Hardy *et al.*, 1979 ▸), the oxidized sulfone, **4**, (KUTDUY; Frampton *et al.*, 1992 ▸) and 4-(4-hy­droxy­phen­yl)-2,2,4-trimethyl-7,8-benzo­thia­chroman **6**, a fused-ring counterpart of *thia*-Dianin’s compound (JELROK; Frampton *et al.*, 2017*c* ▸).

## Synthesis and crystallization   

Preparation of **1**: 4-(4-hy­droxy­phen­yl)-2,2,4-tri­methyl­thia­chroman **3** (MacNicol, 1969 ▸) (0.25 g, 0.88 mmol) was dissolved in glacial acetic acid (10 mL) and a 50% excess of 30% hydrogen peroxide (0.15 mL, 1.32 mmol) added. After the reaction was left overnight at *ca* 278 K, the precipitated white solid was filtered off, washed several times with ether, and initially recrystallized from aqueous dimethyl sulfoxide yielding 0.168 g, (63%) of product. A further recrystallization from glacial acetic acid gave colourless crystals which were analysed by X-ray diffraction as described in the text. The crystals were obtained by spontaneous resolution on crystallization, yielding a 50:50 mixture of the pure enanti­omers. These crystals also incorporated both spontaneously resolved sulfoxide epimers, four further recrystallizations were performed giving a single epimer of purity greater than 99% [500 MHz ^1^H NMR, DMSO-*d_6_* solution analysis gave 99.5 (2)% purity] and the very minor residual second epimer was undetectable in the subsequent X-ray analysis. These crystals melted over a wide range, *ca* 513–536 K, possibly arising from sulfoxide epimerization, along with decomposition, at high temperature. MS [EI^+^]: 300.1178, C_18_H_20_O_2_S, calculated 300.1184; ^1^H NMR (400 MHz, DMSO-*d_6_*) : δ 0.94 (*s*, 3H), 1.31 (*s*, 3H), 1.67 (*s*, 3H), 2.26 (*q*, 2H, δ_AB_ = 0.45 ppm, *J*_AB_ = 15.1 Hz), 6.6–7.7 (aromatic, 8H), 9.27(*s*, 1H); FT–IR (ν_max_, ATR, cm^−1^): 3176 (*br*), 3197 (minor) [ν(O—H)]; 1017 [ν(S—O)].

## Refinement   

Crystal data, data collection and structure refinement details are summarized in Table 2 ▸.

The hydrogen atom of the OH group was localized in the difference-Fourier map and refined isotropically. The other hydrogen atoms were placed in calculated positions and refined within the riding model with C—H = 0.95–0.99 Å and fixed isotropic displacement parameters [*U*_iso_(H) = 1.5*U*_eq_(C) for the methyl groups and 1.2*U*_eq_(C) for the other groups].

## Supplementary Material

Crystal structure: contains datablock(s) I. DOI: 10.1107/S2056989018014366/kq2023sup1.cif

Structure factors: contains datablock(s) I. DOI: 10.1107/S2056989018014366/kq2023Isup2.hkl

Supporting information file. DOI: 10.1107/S2056989018014366/kq2023Isup3.cml

CCDC reference: 1872666

Additional supporting information:  crystallographic information; 3D view; checkCIF report

## Figures and Tables

**Figure 4:**
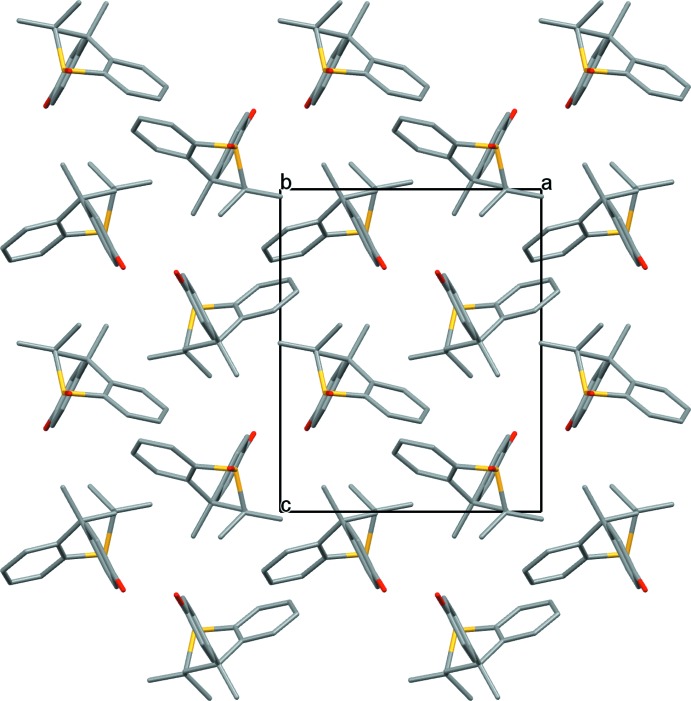
View of the crystal packing of **1** down the *b* axis.

**Table 1:** Hydrogen-bond geometry (Å, °)

*D*—H⋯*A*	*D*—H	H⋯*A*	*D*⋯*A*	*D*—H⋯*A*
O1—H1⋯O2^i^	0.77 (3)	1.92 (3)	2.685 (3)	169 (3)

Symmetry code: (i) 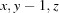.

**Table 2:** Experimental details

Crystal data	
Chemical formula	C_18_H_20_O_2_S
*M*_r_	300.40
Crystal system, space group	Orthorhombic, *P*2_1_2_1_2_1_
Temperature (K)	100
*a*, *b*, *c* (Å)	10.4311 (3), 11.0892 (3), 12.8868 (3)
*V* (Å^3^)	1490.65 (7)
*Z*	4
Radiation type	Cu *K*α
μ (mm^−1^)	1.94
Crystal size (mm)	0.18 × 0.12 × 0.10

Data collection	
Diffractometer	Rigaku Oxford Diffraction SuperNova, Dualflex, AtlasS2
Absorption correction	Gaussian (*CrysAlis PRO*; Rigaku OD, 2015 ▸)
*T*_min_, *T*_max_	0.786, 0.844
No. of measured, independent and observed [*I* > 2σ(*I*)] reflections	5735, 3046, 2982
*R*_int_	0.016
(sin θ/λ)_max_ (Å^−1^)	0.625

Refinement	
*R*[*F* ^2^ > 2σ(*F* ^2^)], *wR*(*F* ^2^), *S*	0.028, 0.075, 1.03
No. of reflections	3046
No. of parameters	197
H-atom treatment	H atoms treated by a mixture of independent and constrained refinement
Δρ_max_, Δρ_min_ (e Å^−3^)	0.27, −0.29
Absolute structure	Flack x determined using 1246 quotients [(*I* ^+^)−(*I* ^−^)]/[(*I* ^+^)+(*I* ^−^)] (Parsons *et al.*, 2013 ▸)
Absolute structure parameter	−0.002 (7)

Computer programs: *CrysAlis PRO* (Rigaku OD, 2015 ▸), *SHELXD2014/6* (Schneider & Sheldrick, 2002 ▸), *SHELXL2014/6* (Sheldrick, 2015 ▸), *SHELXTL* (Sheldrick, 2008 ▸), *Mercury* (Macrae *et al.*, 2008 ▸) and *publCIF* (Westrip, 2010 ▸).

## supplementary crystallographic information

### Crystal data

**Table:**

C_18_H_20_O_2_S	*D*_x_ = 1.339 Mg m^−^^3^
*M**_r_* = 300.40	Cu *K*α radiation, λ = 1.54184 Å
Orthorhombic, *P*2_1_2_1_2_1_	Cell parameters from 3948 reflections
*a* = 10.4311 (3) Å	θ = 5.3–74.2°
*b* = 11.0892 (3) Å	µ = 1.94 mm^−^^1^
*c* = 12.8868 (3) Å	*T* = 100 K
*V* = 1490.65 (7) Å^3^	Block, colourless
*Z* = 4	0.18 × 0.12 × 0.10 mm
*F*(000) = 640	

### Data collection

**Table:**

Rigaku Oxford Diffraction SuperNova, Dualflex, AtlasS2 diffractometer	3046 independent reflections
Radiation source: fine-focus sealed X-ray tube, Enhance (Cu) X-ray Source	2982 reflections with *I* > 2σ(*I*)
Detector resolution: 5.2921 pixels mm^-1^	*R*_int_ = 0.016
ω scans	θ_max_ = 74.5°, θ_min_ = 5.3°
Absorption correction: gaussian (CrysAlisPro; Rigaku OD, 2015)	*h* = −12→13
*T*_min_ = 0.786, *T*_max_ = 0.844	*k* = −12→13
5735 measured reflections	*l* = −16→13

### Refinement

**Table:**

Refinement on *F*^2^	Secondary atom site location: difference Fourier map
Least-squares matrix: full	Hydrogen site location: mixed
*R*[*F*^2^ > 2σ(*F*^2^)] = 0.028	H atoms treated by a mixture of independent and constrained refinement
*wR*(*F*^2^) = 0.075	*w* = 1/[σ^2^(*F*_o_^2^) + (0.0407*P*)^2^ + 0.434*P*] where *P* = (*F*_o_^2^ + 2*F*_c_^2^)/3
*S* = 1.03	(Δ/σ)_max_ = 0.001
3046 reflections	Δρ_max_ = 0.27 e Å^−^^3^
197 parameters	Δρ_min_ = −0.29 e Å^−^^3^
0 restraints	Absolute structure: Flack x determined using 1246 quotients [(*I*^+^)-(*I*^-^)]/[(*I*^+^)+(*I*^-^)] (Parsons *et al.*, 2013)
Primary atom site location: difference Fourier map	Absolute structure parameter: −0.002 (7)

### Special details

**Table:**

Geometry. All esds (except the esd in the dihedral angle between two l.s. planes) are estimated using the full covariance matrix. The cell esds are taken into account individually in the estimation of esds in distances, angles and torsion angles; correlations between esds in cell parameters are only used when they are defined by crystal symmetry. An approximate (isotropic) treatment of cell esds is used for estimating esds involving l.s. planes.

### Fractional atomic coordinates and isotropic or equivalent isotropic displacement parameters (Å^2^)

**Table:**

	*x*	*y*	*z*	*U*_iso_*/*U*_eq_	
S1	0.17202 (5)	0.62246 (5)	0.63136 (4)	0.01942 (13)	
O1	0.10168 (17)	−0.05587 (16)	0.74216 (15)	0.0286 (4)	
H1	0.120 (3)	−0.115 (3)	0.713 (2)	0.031 (8)*	
O2	0.19457 (17)	0.75661 (14)	0.63392 (14)	0.0290 (4)	
C2	0.1359 (2)	0.5775 (2)	0.49805 (16)	0.0213 (5)	
C3	0.1350 (2)	0.4390 (2)	0.50523 (16)	0.0203 (4)	
H3A	0.1008	0.4077	0.4388	0.024*	
H3B	0.0729	0.4164	0.5600	0.024*	
C4	0.2625 (2)	0.3703 (2)	0.52807 (15)	0.0183 (4)	
C5	0.3535 (2)	0.4405 (2)	0.59971 (15)	0.0191 (4)	
C6	0.4737 (2)	0.3919 (2)	0.62341 (17)	0.0229 (4)	
H6	0.4947	0.3139	0.5981	0.027*	
C7	0.5633 (2)	0.4536 (2)	0.68256 (18)	0.0261 (5)	
H7	0.6437	0.4175	0.6977	0.031*	
C8	0.5354 (2)	0.5688 (2)	0.71980 (18)	0.0246 (5)	
H8	0.5973	0.6126	0.7585	0.030*	
C9	0.4163 (2)	0.6183 (2)	0.69968 (15)	0.0211 (4)	
H9	0.3956	0.6962	0.7255	0.025*	
C10	0.3267 (2)	0.55413 (19)	0.64149 (15)	0.0190 (4)	
C11	0.2254 (2)	0.2522 (2)	0.58256 (16)	0.0178 (4)	
C12	0.1659 (2)	0.25810 (19)	0.67999 (16)	0.0195 (4)	
H12	0.1519	0.3348	0.7109	0.023*	
C13	0.1269 (2)	0.1558 (2)	0.73240 (16)	0.0201 (4)	
H13	0.0890	0.1629	0.7991	0.024*	
C14	0.1431 (2)	0.04203 (19)	0.68765 (17)	0.0206 (4)	
C15	0.2012 (2)	0.0339 (2)	0.59057 (18)	0.0224 (4)	
H15	0.2127	−0.0427	0.5589	0.027*	
C16	0.2426 (2)	0.1379 (2)	0.53978 (16)	0.0207 (4)	
H16	0.2836	0.1306	0.4743	0.025*	
C17	0.3331 (2)	0.3456 (2)	0.42483 (16)	0.0237 (4)	
H17A	0.4110	0.2988	0.4386	0.036*	
H17B	0.3563	0.4224	0.3922	0.036*	
H17C	0.2769	0.3000	0.3782	0.036*	
C18	0.2289 (2)	0.6345 (2)	0.42175 (17)	0.0290 (5)	
H18A	0.2208	0.7225	0.4249	0.044*	
H18B	0.2091	0.6067	0.3514	0.044*	
H18C	0.3167	0.6112	0.4398	0.044*	
C19	−0.0004 (2)	0.6226 (2)	0.47748 (17)	0.0261 (5)	
H19A	−0.0595	0.5856	0.5273	0.039*	
H19B	−0.0261	0.6006	0.4068	0.039*	
H19C	−0.0030	0.7105	0.4851	0.039*	

### Atomic displacement parameters (Å^2^)

**Table:**

	*U*^11^	*U*^22^	*U*^33^	*U*^12^	*U*^13^	*U*^23^
S1	0.0224 (2)	0.0180 (2)	0.0179 (2)	0.0006 (2)	−0.00050 (19)	−0.00088 (18)
O1	0.0335 (9)	0.0175 (8)	0.0347 (9)	−0.0018 (7)	0.0080 (7)	0.0026 (7)
O2	0.0354 (9)	0.0181 (7)	0.0335 (9)	0.0019 (7)	−0.0041 (8)	−0.0025 (7)
C2	0.0268 (11)	0.0212 (11)	0.0159 (9)	0.0007 (8)	−0.0021 (8)	0.0008 (8)
C3	0.0235 (10)	0.0205 (10)	0.0169 (9)	0.0001 (9)	−0.0028 (8)	0.0003 (8)
C4	0.0214 (9)	0.0186 (10)	0.0150 (9)	−0.0011 (9)	0.0001 (7)	−0.0018 (9)
C5	0.0210 (10)	0.0217 (10)	0.0146 (8)	−0.0036 (8)	0.0026 (7)	0.0013 (8)
C6	0.0229 (10)	0.0221 (10)	0.0238 (10)	0.0015 (8)	0.0011 (8)	−0.0022 (9)
C7	0.0215 (10)	0.0308 (12)	0.0261 (11)	0.0015 (9)	0.0001 (9)	0.0005 (10)
C8	0.0215 (10)	0.0292 (12)	0.0231 (10)	−0.0053 (9)	−0.0024 (8)	−0.0021 (9)
C9	0.0263 (10)	0.0194 (10)	0.0177 (9)	−0.0020 (9)	0.0006 (8)	0.0000 (8)
C10	0.0200 (9)	0.0218 (9)	0.0151 (8)	−0.0032 (9)	0.0014 (9)	0.0028 (7)
C11	0.0183 (9)	0.0186 (10)	0.0166 (9)	0.0001 (8)	−0.0020 (8)	−0.0009 (8)
C12	0.0202 (9)	0.0188 (9)	0.0194 (9)	−0.0016 (9)	−0.0008 (9)	−0.0034 (8)
C13	0.0199 (9)	0.0217 (11)	0.0188 (9)	−0.0009 (8)	0.0019 (8)	−0.0003 (8)
C14	0.0187 (10)	0.0184 (10)	0.0247 (10)	−0.0017 (8)	−0.0017 (8)	0.0023 (9)
C15	0.0258 (11)	0.0168 (10)	0.0246 (10)	0.0005 (8)	−0.0016 (9)	−0.0039 (9)
C16	0.0236 (10)	0.0193 (10)	0.0191 (9)	0.0006 (9)	−0.0001 (8)	−0.0016 (9)
C17	0.0296 (11)	0.0253 (11)	0.0161 (9)	0.0002 (9)	0.0033 (9)	−0.0008 (7)
C18	0.0387 (13)	0.0280 (12)	0.0204 (10)	−0.0003 (11)	0.0040 (10)	0.0048 (10)
C19	0.0291 (11)	0.0270 (12)	0.0220 (10)	0.0052 (11)	−0.0055 (8)	0.0010 (10)

### Geometric parameters (Å, º)

**Table:**

S1—O2	1.5064 (16)	C9—C10	1.394 (3)
S1—C10	1.788 (2)	C9—H9	0.9500
S1—C2	1.828 (2)	C11—C16	1.394 (3)
O1—C14	1.363 (3)	C11—C12	1.402 (3)
O1—H1	0.77 (3)	C12—C13	1.381 (3)
C2—C18	1.519 (3)	C12—H12	0.9500
C2—C19	1.530 (3)	C13—C14	1.398 (3)
C2—C3	1.539 (3)	C13—H13	0.9500
C3—C4	1.560 (3)	C14—C15	1.393 (3)
C3—H3A	0.9900	C15—C16	1.395 (3)
C3—H3B	0.9900	C15—H15	0.9500
C4—C11	1.535 (3)	C16—H16	0.9500
C4—C5	1.536 (3)	C17—H17A	0.9800
C4—C17	1.545 (3)	C17—H17B	0.9800
C5—C10	1.398 (3)	C17—H17C	0.9800
C5—C6	1.399 (3)	C18—H18A	0.9800
C6—C7	1.387 (3)	C18—H18B	0.9800
C6—H6	0.9500	C18—H18C	0.9800
C7—C8	1.395 (4)	C19—H19A	0.9800
C7—H7	0.9500	C19—H19B	0.9800
C8—C9	1.383 (3)	C19—H19C	0.9800
C8—H8	0.9500		
			
O2—S1—C10	106.02 (10)	C9—C10—S1	115.34 (17)
O2—S1—C2	108.79 (10)	C5—C10—S1	122.27 (16)
C10—S1—C2	98.01 (10)	C16—C11—C12	117.0 (2)
C14—O1—H1	110 (2)	C16—C11—C4	124.22 (19)
C18—C2—C19	110.19 (19)	C12—C11—C4	118.76 (19)
C18—C2—C3	117.26 (19)	C13—C12—C11	122.0 (2)
C19—C2—C3	109.35 (18)	C13—C12—H12	119.0
C18—C2—S1	111.29 (16)	C11—C12—H12	119.0
C19—C2—S1	105.39 (15)	C12—C13—C14	120.26 (19)
C3—C2—S1	102.53 (14)	C12—C13—H13	119.9
C2—C3—C4	119.60 (18)	C14—C13—H13	119.9
C2—C3—H3A	107.4	O1—C14—C15	123.3 (2)
C4—C3—H3A	107.4	O1—C14—C13	117.9 (2)
C2—C3—H3B	107.4	C15—C14—C13	118.8 (2)
C4—C3—H3B	107.4	C14—C15—C16	120.2 (2)
H3A—C3—H3B	107.0	C14—C15—H15	119.9
C11—C4—C5	108.25 (16)	C16—C15—H15	119.9
C11—C4—C17	111.28 (18)	C11—C16—C15	121.8 (2)
C5—C4—C17	108.20 (17)	C11—C16—H16	119.1
C11—C4—C3	106.76 (17)	C15—C16—H16	119.1
C5—C4—C3	113.09 (18)	C4—C17—H17A	109.5
C17—C4—C3	109.29 (17)	C4—C17—H17B	109.5
C10—C5—C6	116.23 (19)	H17A—C17—H17B	109.5
C10—C5—C4	124.4 (2)	C4—C17—H17C	109.5
C6—C5—C4	119.3 (2)	H17A—C17—H17C	109.5
C7—C6—C5	122.3 (2)	H17B—C17—H17C	109.5
C7—C6—H6	118.9	C2—C18—H18A	109.5
C5—C6—H6	118.9	C2—C18—H18B	109.5
C6—C7—C8	120.0 (2)	H18A—C18—H18B	109.5
C6—C7—H7	120.0	C2—C18—H18C	109.5
C8—C7—H7	120.0	H18A—C18—H18C	109.5
C9—C8—C7	119.2 (2)	H18B—C18—H18C	109.5
C9—C8—H8	120.4	C2—C19—H19A	109.5
C7—C8—H8	120.4	C2—C19—H19B	109.5
C8—C9—C10	120.0 (2)	H19A—C19—H19B	109.5
C8—C9—H9	120.0	C2—C19—H19C	109.5
C10—C9—H9	120.0	H19A—C19—H19C	109.5
C9—C10—C5	122.3 (2)	H19B—C19—H19C	109.5
			
O2—S1—C2—C18	45.94 (19)	C6—C5—C10—C9	2.8 (3)
C10—S1—C2—C18	−64.08 (18)	C4—C5—C10—C9	−175.00 (19)
O2—S1—C2—C19	−73.49 (17)	C6—C5—C10—S1	−172.93 (15)
C10—S1—C2—C19	176.50 (15)	C4—C5—C10—S1	9.3 (3)
O2—S1—C2—C3	172.10 (14)	O2—S1—C10—C9	31.70 (18)
C10—S1—C2—C3	62.09 (15)	C2—S1—C10—C9	143.96 (16)
C18—C2—C3—C4	54.9 (3)	O2—S1—C10—C5	−152.32 (17)
C19—C2—C3—C4	−178.82 (18)	C2—S1—C10—C5	−40.06 (19)
S1—C2—C3—C4	−67.3 (2)	C5—C4—C11—C16	−124.3 (2)
C2—C3—C4—C11	152.40 (17)	C17—C4—C11—C16	−5.6 (3)
C2—C3—C4—C5	33.5 (2)	C3—C4—C11—C16	113.6 (2)
C2—C3—C4—C17	−87.1 (2)	C5—C4—C11—C12	58.2 (2)
C11—C4—C5—C10	−117.8 (2)	C17—C4—C11—C12	176.96 (19)
C17—C4—C5—C10	121.5 (2)	C3—C4—C11—C12	−63.9 (2)
C3—C4—C5—C10	0.3 (3)	C16—C11—C12—C13	0.7 (3)
C11—C4—C5—C6	64.5 (2)	C4—C11—C12—C13	178.3 (2)
C17—C4—C5—C6	−56.2 (2)	C11—C12—C13—C14	−1.7 (3)
C3—C4—C5—C6	−177.41 (19)	C12—C13—C14—O1	−179.3 (2)
C10—C5—C6—C7	−1.7 (3)	C12—C13—C14—C15	1.2 (3)
C4—C5—C6—C7	176.2 (2)	O1—C14—C15—C16	−179.3 (2)
C5—C6—C7—C8	−0.6 (4)	C13—C14—C15—C16	0.3 (3)
C6—C7—C8—C9	2.0 (4)	C12—C11—C16—C15	0.8 (3)
C7—C8—C9—C10	−1.0 (3)	C4—C11—C16—C15	−176.7 (2)
C8—C9—C10—C5	−1.5 (3)	C14—C15—C16—C11	−1.3 (3)
C8—C9—C10—S1	174.49 (17)		

### Hydrogen-bond geometry (Å, º)

**Table:**

*D*—H···*A*	*D*—H	H···*A*	*D*···*A*	*D*—H···*A*
O1—H1···O2^i^	0.77 (3)	1.92 (3)	2.685 (3)	169 (3)

Symmetry code: (i) *x*, *y*−1, *z*.
